# Suppressed RNA-Polymerase 1 Pathway Is Associated with Benign Multiple Sclerosis

**DOI:** 10.1371/journal.pone.0046871

**Published:** 2012-10-12

**Authors:** Anat Achiron, Anna Feldman, David Magalashvili, Mark Dolev, Michael Gurevich

**Affiliations:** Multiple Sclerosis Center, Neurogenomics Laboratory, Sheba Medical Center, Tel-Hashomer and Sackler School of Medicine, Tel-Aviv University, Tel Aviv, Israel; Institute Biomedical Research August Pi Sunyer (IDIBAPS) - Hospital Clinic of Barcelona, Spain

## Abstract

Benign multiple sclerosis (BMS) occurs in about 15% of patients with relapsing-remitting multiple sclerosis (RRMS) that over time do not develop significant neurological disability. The molecular events associated with BMS are not clearly understood. This study sought to underlie the biological mechanisms associated with BMS. Blood samples obtained from a cohort of 31 patients with BMS and 36 patients with RRMS were applied for gene expression microarray analysis using HG-U133A-2 array (Affymetrix). Data were analyzed by Partek and pathway reconstruction was performed by Ingenuity for the most informative genes (MIGs). We identified a differing gene expression signature of 406 MIGs between BMS patients, mean±SE age 44.5±1.5 years, 24 females, 7 males, EDSS 1.9±0.2, disease duration 17.0±1.3 years, and RRMS patients, age 40.3±1.8 years, 24 females, 12 males, EDSS 3.5±0.2, disease duration 10.9±1.4 years. The signature was enriched by genes related RNA polymerase I (POL-1) transcription, general inflammatory response and activation of cell death. The most significant under-expressed pathway operating in BMS was the POL-1 pathway (p = 4.0*10^−5^) known while suppressed to activate P53 dependent apoptosis and to suppress NFκB induced inflammation. In accordance, of the 30 P53 target genes presented within the BMS signature, 19 had expression direction consistent with P53 activation. The transcripts within the pathway include POL-1 transcription factor 3 (RRN3, p = 4.8*10^−5^), POL-1 polypeptide D (POLR1D, p = 2.2*10^−4^), leucine-rich PPR-motif containing protein (LRPPRC p = 2.3*10^−5)^, followed by suppression of the downstream family of ribosomal genes like RPL3, 6,13,22 and RPS6. In accordance POL-1 transcript and release factor PTRF that terminates POL-1 transcription, was over-expressed (p = 4.4*10^−3^). Verification of POL-1 pathway key genes was confirmed by qRT-PCR, and RRN3 silencing resulted in significant increase in the apoptosis level of PBMC sub-populations in RRMS patients. Our findings demonstrate that suppression of POL-1 pathway induce the low disease activity of BMS.

## Introduction

Multiple sclerosis (MS), the most common demyelinating disease of the central nervous system (CNS) affecting young adults is subdivided into several clinical subtypes; the most common disease course is relapsing-remitting MS (RRMS) that occurs in 85% of patients; it is characterized by acute attacks manifested with various neurological symptoms including varying combinations of motor, sensory, coordination, visual, and cognitive impairments, as well as symptoms of fatigue and urinary tract dysfunction [1]. It is well established that with each attack, the probability of complete clinical remission decreases, and neurological disability and handicap are liable to develop. However, in about 15% of RRMS patients the disease has a benign course, in which patients remain fully functional for a period longer than 10 or 15 years after onset as demonstrated by low neurological disability with an Expanded Disability Status Scale (EDSS) score lower than 3.0 [2]–[6]. These patients with benign MS (BMS) signify a group of non-active patients, in whom in spite of the on-going disease process, patients are protected from disability. It is not yet clear what may lead to a benign course in some patients, while in others the disease has malignant progression with many relapses and significant disability within a short period of time. Patients with BMS reflect either the capacity to withstand the deteriorating processes of the disease and the ability to endure or recover quickly from the acute demyelinating inflammatory insult as well as not to be harmed by the ongoing pathologic process of neuronal and myelin loss, or these patients may have mild disease. A comprehensive knowledge of the biological mechanisms operating in BMS is of importance as it could lead to the identification of innovative targets associated with modulation of inflammation to reduce MS disease activity.

Previous studies using peripheral blood gene expression arrays characterized MS pathogenesis, progression and response to treatment [7]–[10].

In the current study we aimed to characterize the expression profile of BMS in comparison to typical RRMS using high throughput gene expression microarrays, in order to identify compensating biological mechanisms that are particularly effective to prevent neuronal damage and maintain low disease activity. We further constructed molecular regulatory networks and identified Polymerase-I molecular pathway as a potential target for therapeutic interventions.

## Materials and Methods

### Study design

For the purpose of this study the Sheba Multiple Sclerosis computerized database was queried for eligible patients according to the following criteria. Inclusion criteria: (1) Relapsing-remitting disease course; (2) Low neurological disability by the Expanded Disability Status Scale (EDSS) of score less than 3.0 within 15 years or more after disease onset for the BMS group according to the international survey by the USA National MS Society [11], or EDSS score greater than 3.0 within less than 15 years after disease onset for the RRMS group; Exclusion criteria: (1) acute relapse; (2) treatment with corticosteroids within 6 months prior to the study; (3) Pregnancy. Eligible patients were asked to give a blood sample for the gene expression analysis. The study was approved by Sheba IRB Ethical Committee and all patients signed written informed consent.

### Gene expression analyses

Peripheral blood mononuclear cells (PBMC) were separated on ficoll-hypaque gradient. Total RNA was isolated using the TRIzol Reagent (Invitrogen, Carlsbad, CA). RNA quality was assessed using Experion Automated Electrophoresis Station (Bio-Rad) and quantified by fiberoptic spectrophotometry using the Nanodrop ND-1000 (Nanodrop Inc., Wilmington, DE, USA). cDNA was synthesized, labeled and hybridized to HG-U133A-2 array (Affymetrix, Inc., Santa Clara, CA) containing 22,215 gene-transcripts (corresponded to 14500 well characterized human genes), washed and scanned (Hewlett Packard, GeneArray-TM scanner G2500A) according to manufacturer's protocol Affymetrix (Inc., Santa Clara, CA).

### Data analysis

Data analysis was performed using the Partek Genomics Solution software (www.partek.com). For the row data normalization we have applied the Robust Multichip Average (RMA) method. This method consists of three steps: a background adjustment, quantile normalization and final summarization, of which the background correction is the most crucial step for probe level processing and is performed on a per-array basis. The quantile normalization step was applied to make the distributions identical across arrays. Once the probe-levels values have been background-corrected and normalized, they are summarized into expression measures, so that the result is a single expression measure per probe-set, per array. We used log transformation and median polishing as a robust model fitting technique that protects against outlier probes.

As the microarrays were produced by several working sets, the RMA procedure ran several times on different sets of arrays. In order to avoid the noise caused by variable set effect, we performed quintile normalization of all batches to the same distribution of a well balanced set used as a reference distribution that enabled comparable signals of all arrays.

The Batch RemoverTM algorithm based on mixed model ANOVA implemented in Partek software was used to analyze and reduce nuisance confounders' effects such as age, gender and immunomodulatory treatment. After the batch effect was removed the variables were re-included in the ANOVA model to account for the appropriate degrees of freedom and to correct the p values calculations.

Most informative genes (MIGs) were defined as genes with p<0.01 by ANOVA linear contrasts model.

### Functional annotations of MIGs

Gene functional annotations and enrichment and pathway analysis were performed by Gene Ontology (GO), term enrichment analysis based on DAVID Functional Annotation Bioinformatics Resources v6, Kyoto Encyclopedia of Genes and Genomes (KEGG), PANTHER and Ingenuity Pathway Analysis® suite. IPA web software was also used to reconstruct gene and transcriptional factors functional networks. All p values were applied for multiple testing corrections using Benjamini- Hochberg FDR method with a cut off at p = 0.05.

### Verification of POL-1 pathway key genes by q-RT-PCR

POL-1 pathway key genes POLR1D, RRN3 (p = 0.03) and LRPPRC were verified by quantitative RT-PCR on 7500 Real-time PCR Cycler (Applied Biosystem, CA, USA) using Taqman probes adjusted to each of the following genes: RRN3-HS00607907_m1, POLR1D- HS00211081_m1 and LRPPRC – HS00211081 m1. Analysis was carried out by 7500 System W1.2.2 software. Relative quantification values were calculated using ΔCT method. The house keeping gene GAPDH expression levels were used as internal control for sample normalization. Verification experiments were performed in blood samples obtained from an additional cohort of 35 MS patients; 20 BMS patients (mean+SE age 46.9±2.1 years, 15 females, 5 males, disease duration 18.1±1.6 years, EDSS 1.9±0.3) and 15 RRMS patients (age 41.5±2.8 years, 11 females, 4 males, disease duration 7.9±1.2 years, EDSS 4.4±0.3). T-test was used to calculate statistical significance.

### Evaluation of RRN3 effect on apoptosis

#### (1) Apoptosis assay

PBMC were isolated from 12 MS patients (6 patients with BMS, and 6 patients with RRMS) using Ficoll-Hypaque (Invitrogen, USA) gradient.

FITC-conjugated magnetic beads BD-IMAGTM (BD bioscience, USA) kit was used for CD4^+^, CD8^+^, CD14^+^, CD19^+^ and CD56^+^ cell isolation. Apoptosis level was measured by flow cytometery (FACS, Becton Dickinson, USA) using FITC/propidium iodide (PI, 0.5 mg/ml, Sigma- Aldrich, USA). Cells were stained and the percentage of apoptotic cells was calculated out of the total cell count. Fluorescence intensity measurements were performed after excitation at 488 nm and detection at 520–530 nm for green fluorescence and at 665–685 nm for PI.

#### (2) RRN3 silencing

To confirm the role of Pol- 1 pathway on the mechanism of apoptosis in RRMS, we performed post-transcriptional silencing of the key RRN3 gene by siRNA and than tested for the percent of apoptotic cells by FACS. RRN3 gene silencing was performed using RRN3 siRNA Plasmid kit (sc-153128-SH, Santa Cruz biotechnology, Inc., CA) in 6-well tissue culture plate according to manufacturer's instructions. siRNA-A (scrambled sequence that does not lead to degradation of the target gene mRNA, SCI, USA) was used as negative control. The knockdown efficiency was confirmed by Western Blot using goat polyclonal RRN3 (E-20) antibody (Santa Cruz, USA) in PBMC lysates. Recombinant RRN3 protein (Santa Cruz, USA) was used as a positive control.

## Results

### Subjects

The Sheba MS Center database includes the data of 2200 patients with RRMS, of these 720 patients have a follow-up period since onset of at least 15 years, and of these 12.6% (91 patients) met the diagnostic criteria of BMS with EDSS< = 3.0. For the purpose of the current study 31 BMS patients, 24 females, 7 males, mean age 44.5±1.5 years, with low disability as was evident by mean EDSS score of 1.9±0.15, low annual EDSS increase of 0.13±0.01, and low annual relapse rate of 0.23±0.04, with long disease duration of 17.0±1.3 years, were included. The gene expression data of the BMS patients were compared with 36 RRMS patients, 24 females, 12 males, mean age 40.3±1.8 years, characterized by a mean EDSS score of 3.5±0.23, with a mean annual EDSS increase of 0.45±0.06, and annual relapse rate of 0.64±0.09, through a disease duration of 10.9±1.4 years. Twelve out of the 31 BMS patients (38.7%) and 18 of the 36 RRMS patients (50%) were treated with immunomodulatory treatments during blood withdrawal.

### Differential expression between BMS and RRMS patients

**Figure 1:**
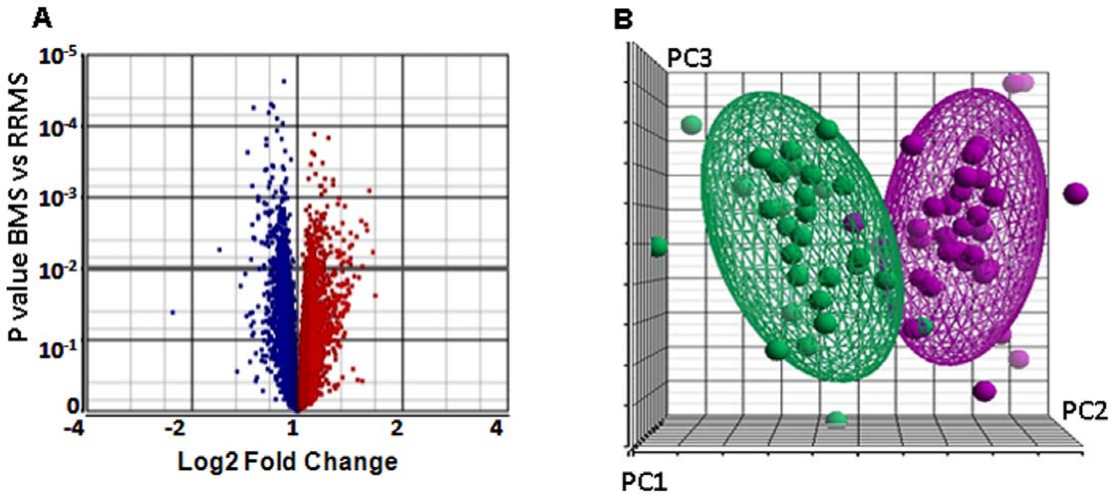
Differential expression between BMS and RRMS. A. Volcano plot based on all microarray transcripts demonstrates global p value and Log2 fold change for each gene in differentiating between PBMC gene expression of patients with BMS and patients with RRMS. Red dots display over-expressed genes, blue dots display down-expressed genes. 406 MIGs, 171 gene over-expressed and 235 down-expressed, with p<0.01 and a log fold change between -3.1 to 3.3, are demonstrated above the black horizontal line. B. Principal component analysis (PCA) plot for microarray data showing the difference between BMS and RRMS blood gene expression. The three first principal components PC1, PC2 and PC3 are the linear combinations of the expressions of 406 MIGs plotted with the proportion of variance explained by each component, which covered 70.0% of total variance. The different ellipsoids plotted in 3-dimentional space show clear separation between BMS (violet dots, N = 31) and RRMS (green dots, BMS = 36) patients.

The statistical scoring analysis applied to the microarray data demonstrated that BMS and RRMS peripheral blood gene expression signatures were different by 406 MIGs; 171 gene transcripts were over-expressed and 235 were down-expressed, with a log fold change that ranged from −3.1 to 3.3, Fig. 1A, B, Table S1.

The BMS signature was enriched by genes related RNA polymerase I (POL-1) transcription (p = 0.01), mRNA transcription initiation (p = 0.07), ribosome (p = 0.07), translational elongation (2.5*10^−4^), transcription initiation (p = 0.001) and regulation of mitotic cell cycle (p = 0.001). Additional important biological processes that were enriched were related to general inflammatory responses such as regulation of lymphocyte activation (p = 0.001), cytokine and chemokine mediated signaling (p = 0.018), T-cell mediated immunity (p = 0.026) and adherens junction (p = 0.04), and activation of cell death (p =  5.5*10^−7^ to 1.6*10^−2^) that was enriched by genes related to initial stage of apoptosis like FAS, execution-phase of cell apoptosis like CASP10 and over-expressed TOSO - negative regulator of apoptosis. The group of genes encoding death associated transcription factors like DATF1 and proteins such as DDX42 as well as 30 P53-downstream genes, further confirm the activation of apoptotic mechanisms.

**Figure 2:**
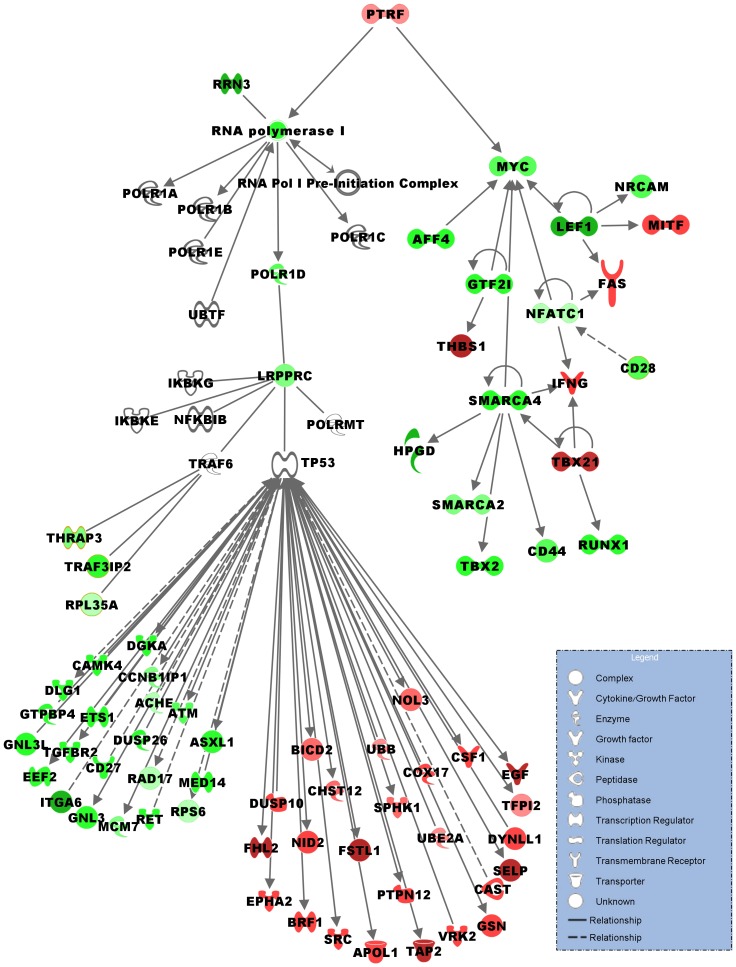
Gene-expression regulatory network of POL-1 pathway in BMS. Pathway analysis by Ingenuity software based on BMS 406 related MIG. The relations between the genes were inferred from the relationships known in the scientific literature using data-mining Ingenuity software. Each node represents a gene; red color denotes over-expressed genes; green color denotes down-expressed genes. The colors intensity appears according to the related expression level by fold change. Connections indicate regulatory interactions as follows: arrows = direct activation; dashed arrows = indirect activation.

Transcription factor (TFs) analysis of BMS signature predicted 49 that potentially could regulate the corresponding target genes. From those, 7 TFs including LEF1, NFATC1, AFF4, GTF21, SMARCA4, PTRF and TBX21 indeed changed their transcription level in BMS as compared to RRMS patients, Fig. 2, Table S1. An interesting finding is the over-expression of PTRF TF that functions as POL-1 and transcript release factor able to bind to POL-1 complex and pause it transcription. This result confirms the findings of the enrichment analysis that pointed to POL-1 as the most significant pathway in the BMS signature using PANTHER database. Two important T cell related TFs that play a central role in inducible gene transcription during immune response were down-regulated in BMS; LEF1 TF that encodes protein binding to a T-cell receptor-alpha enhancer and thereby confers maximal enhancer activity, together with NFATC1, a component of the nuclear factor of activated T cells that translocates to the nucleus upon T cell receptor stimulation, and SMARCA4 TF known to be involved in regulation of CD44. Interestingly, 5 of the 7 TFs regulate MYC target gene that operates as transcription regulator.

P53 TF did not change its transcription level in the BMS signature, however it was found as the most significant TF (z-score = 2.20) that was predicted to be activated. Of the 30 P53 target genes presented within the BMS signature, 19 had expression direction consistent with P53 activation, BMS related transcription factor network is presented in Fig. S1.

### RNA-polymerase-I pathway

The most significant under-expressed pathway operating in BMS was the RNA-polymerase-I (POL-1) pathway (p = 4.0*10^−5^) that is known while suppressed to activate P53 dependent apoptosis and to suppress NFKB induced inflammation. The significant transcripts within the pathway include POL-1 transcription factor 3 (RRN3, p = 4.8*10^−5^), polymerase RNA I polypeptide D (POLR1D, p = 2.2*10^−4^), leucine-rich PPR-motif containing protein (LRPPRC p = 2.3*10^−5^), followed by the downstream family of ribosomal genes like RPL3, RRPL6, RRPL13, RRPL22, RRPL35A, RPS6, and EIF3, Table 1. In accordance, the suppression of POL-1 regulation mechanism was confirmed by over-expression of POL-1 transcript and release factor (PTRF, p = 4.4*10^−3^) that terminates POL-1 transcription, while the activation of P53 pathway was evidenced by over-expression of TP53TG3 and TP53AP1 P53 inducible genes. The gene-expression regulatory network of POL-1 pathway in BMS is demonstrated in Fig. 2.

**Table 1:** POL-1 pathway related genes: BMS vs. RRMS.

Gene Symbol	Gene Title	p-value BMS vs. RRMS	Fold Change BMS/RRMS
RRN3	RRN3 RNA polymerase I transcription factor homolog (yeast)	4.86E-05	−2.28514
LRPPRC	leucine-rich PPR-motif containing	2.30E-05	−2.13048
POLR1D	polymerase (RNA) I polypeptide D, 16 kDa	2.71E-03	−2.17804
PTRF	polymerase I and transcript release factor	4.43E-03	2.088823
MYC	v-myc myelocytomatosis viral oncogene homolog (avian)	7.39E-03	−2.22632
RPL3	ribosomal protein L3///ribosomal protein L3	6.17E-03	−2.07132
RPL6	ribosomal protein L6///ribosomal protein L6	9.34E-03	−2.0569
RPS6	ribosomal protein S6///ribosomal protein S6	1.02E-03	−2.0905
RPL22	ribosomal protein L22	3.11E-03	−2.1251

### Verification

**Figure 3:**
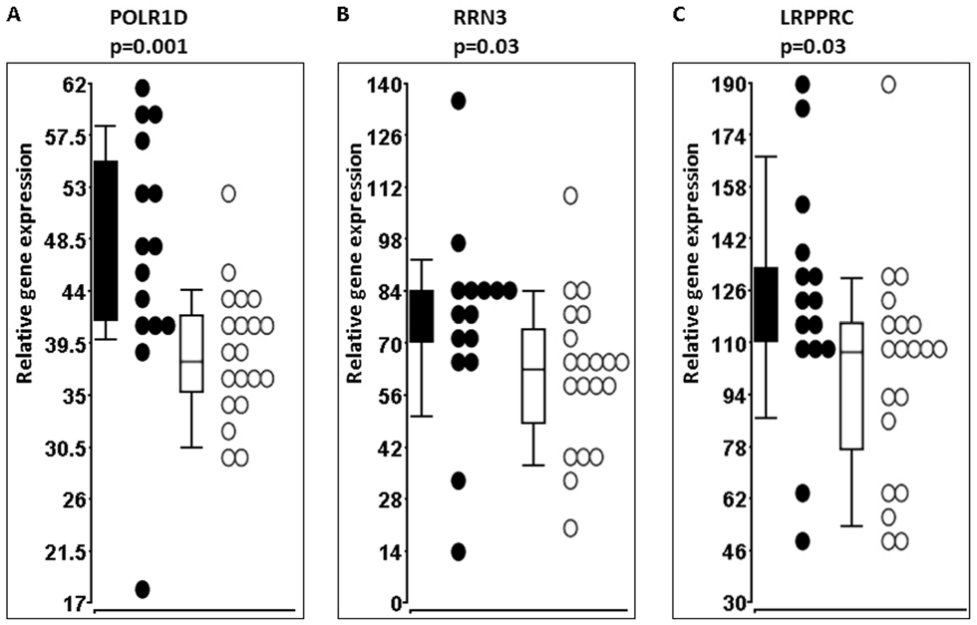
POL-1 pathway key genes verification by qRT-PCR. White dots represent BMS patients (N = 20), black dots represent RRMS patients (N = 15). Data are presented as relative quantification values using ΔCT method. The house keeping gene GAPDH expression levels were used as internal control for sample normalization. Low level of the POL-1 pathway key genes POLR1D (p = 0.001), RRN3 (p = 0.03) and LRPPRC (p = 0.03) is demonstrated in BMB patients as compared with RRMS patients.

Verification of POL-1 pathway key genes by qRT-PCR in 20 BMS and 15 RRMS patients confirmed the microarray findings. Significantly low levels of POLR1D (p = 0.001), RRN3 (p = 0.03) and LRPPRC (p = 0.03) were demonstrated in BMS patients as compared with RRMS patients, Fig. 3.

### Effect of RRN3 on apoptosis

**Figure 4:**
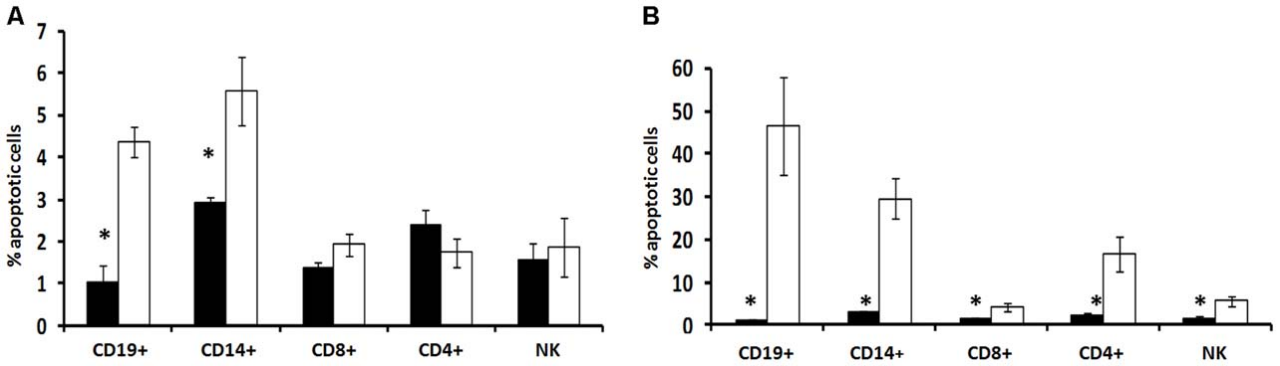
Effect of RRN3 silencing on apoptosis in RRMS. A. Comparison of apoptotic level in PBMC sub-populations between BMS (white bars) and RRMS (black bars) patients. A significantly higher percent of apoptotic CD19+ B cells and CD14+ macrophages is demonstrated in BMS patients. B. Apoptosis level in PBMC sub-populations of RRMS patients before and after RRN3 silencing. Percent of apoptotic cells was measured by PI staining.

Comparison of apoptotic level in PBMC subtypes between BMS and RRMS patients demonstrated higher level of apoptotic cells in BMS patients and significantly higher percentage of apoptotic CD19+ B cells (p =  0.0029) and CD14+ macrophages (p =  0.0274), Fig. 4A. RRN3 silencing resulted in significant increase in the apoptosis level of PBMC from RRMS patients, evident for all cell types as follows: CD19+ (p = 0.0084), CD14+ (0.0024), CD8+ (p = 0.0167), CD4+ (0.0123) and CD56+NK cells (p = 0.016), Fig. 4B.

## Discussion

In the present study we identified the controlling regulatory networks that differentiate BMS from RRMS. We found that suppression of POL-1 pathway is associated with low threshold to develop active disease. The clinical difference between BMS and RRMS is significant in terms of progression to disability and the relatively higher activity of POL-1 pathway in RRMS is associated with aberrant apoptosis.

The role of ribosomal RNA network in the brain has been shown in cultured cortical neurons where inhibition of ribosomal RNA transcription up-streamed pro-apoptotic p53 activity. Moreover, siRNA-mediated inhibition of RRN3, the RNA POL-1 co-factor, attenuated ribosomal RNA transcription causing nucleolar stress and p53-dependent neuronal apoptosis [12]. In has been also demonstrated that inhibition of RNA-POL-1 mediated ribosomal transcription in RRN3−/− mice leads to activation of P53 [13].

**Figure 5:**
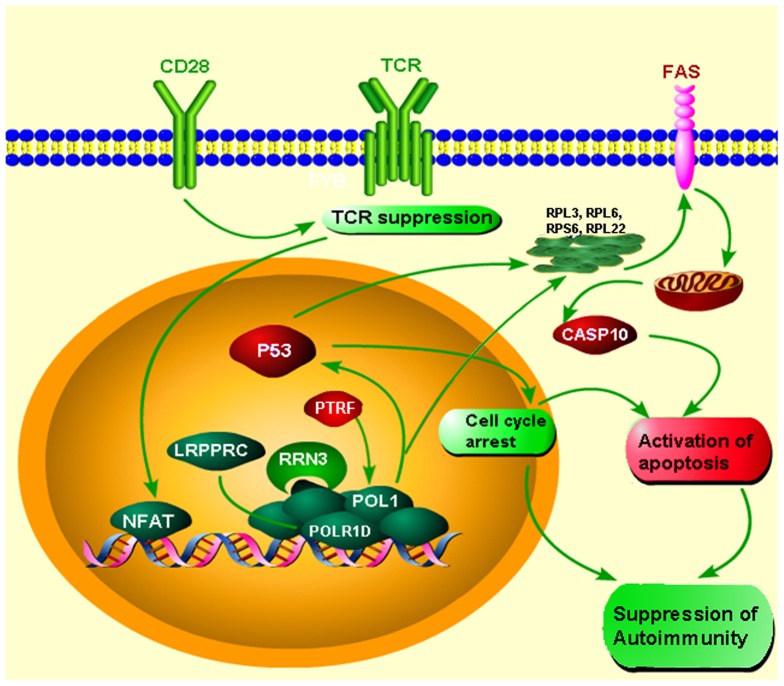
Suppressed POL-1 pathway activity in BMS. A schematic model demonstrating the suppressed POL-1 pathway activity in BMS leading to activation of P53 dependent apoptosis. Over-expressed genes are depicted in red, down-expressed genes in green.

Apoptosis can be regulated by cellular signals that are communicated by ribosomal proteins which have important role in cell proliferation. The anti-survival effects resulting from the down-activation of POL-1 pathway-related genes are transduced by the over-activation of PTRF TF and by the suppression the ribosomal proteins RPL3, RPL6, RPS6 and RPL22, that together increased the expression of P53 and FAS subsequently to activate apoptosis of autoimmune cells and decrease disease activity. The down-expression of the nuclear factor of activated T-cells (NFAT), a transcription factor in activated T cells [14], [15] together with under-expression of co-stimulatory CD28, signal suppression of T cell inflammation in BMS and may also contribute to P53 pro-apoptotic activity [16], Fig. 5. This was further evidenced by a large number of P53 inducible genes and the significant prediction of P53 TFs within the BMS signature.

POL-1 pathway associated genes are dedicated to transcription of rRNA genes. RRN3, a 72-kDa protein, is essential and limiting factor for ribosomal DNA transcription and acts as a bridge between RNA POLI and the committed promoter [17]; LRPPRC plays a role in translation and stability of mitochondrially encoded cytochrome c oxidase (COX) subunits [18], and together with POLR1D comprise a complex with the nuclear factor of kappa light polypeptide gene enhancer in B-cells inhibitor, beta (NFkBIB) that inhibits pro-inflammatory NFkB pathway [19]. Taken together, our findings suggest that low activity of the POL-1 pathway associated genes is associated with a benign course of MS and that the key response to limit MS disease activity is the induction of cell death forcing self-derived autoimmune cells to disappear. These results are in accordance with our previous findings related to the characterization of the gene expression signature for good outcome in MS [20]. We have reported that RRN3 is the most predictive gene accounting for 70.4% of the prediction. In the current study we further demonstrated that blocking RRN3 expression by siRNA-silencing in PBMC cultures of RRMS patients resulted in significant increase in apoptosis confirming that signaling through POL-1 pathway plays a key role in regulating cell survival in MS.

The suggested functional involvement of POL-1 in MS is supported by studies that evaluated the role of two compounds that suppress POL-1 activity in the experimental animal model of autoimmune encephalomyelitis (EAE). The first of these compounds, triptolide (TPT, diterpenoid triepoxide), has immunosuppressive properties and though toxic is effective in prolonging graft survival and suppressing autoimmune responses, was reported to significantly delay EAE onset and severity EAE [21]. The second compound, Rapamycin, a macrocyclic triene antibiotic also with antitumor and immunosuppressive activities was reported to down-regulate POL-1 activity through inhibition of mTOR pathway and to ameliorate the clinical and histological signs of chronic and acute EAE [22]–[25]. mTOR was reported to bind to the promoter region of POL-1 and POL-3 transcribed genes [26], and its inactivation leads to proteasome-dependent degradation of RRN3 [27].

Our findings support a basis for direct targeting of POL-1 transcription pathway as a strategy for selective induction of apoptosis in MS and pave the way for an entirely new class of therapeutic approach to induce transition from the pattern of an active to benign disease. In addition, a possible clinical implication of the obtained results is the possibility to monitor disease activity in MS patients and identify those who will have a benign or a more active relapsing course. As the gene expression profiles and pathways identified were not obtained prospectively, further studies are needed to clarify the effects of which specific cell-type express the BMS signature and how it is related to the target tissue.

## Supporting Information

Table S1 **List of the 406 MIGs differentiating between BMS and RRMS.**
(XLS)

Figure S1 **Transcription factors and corresponding genes operating in BMS.** The relations between TFs and corresponding genes within the BMS signature were inferred using data-mining IPA software. The inner circle represents 49 predicted TFs that potentially could regulate the corresponding target genes within the BMS signature, represented in the external circle. Colored nodes present MIGs or TFs that significantly changed within the signature; red – over-expressed, green – down-expressed; white nodes represent TFs that were predicted (z-score) to be involved in the target genes regulation, while only seven colored TFs indeed changed their expression level within the BMS signature.
(TIF)
