# Supplementary figures and images for: Suppressed RNA-Polymerase 1 Pathway Is Associated with Benign Multiple Sclerosis
Source: PLoS One. 2012 Oct 12;7(10):e46871. doi: 10.1371/journal.pone.0046871 (PMC3470584; doi:10.1371/journal.pone.0046871)

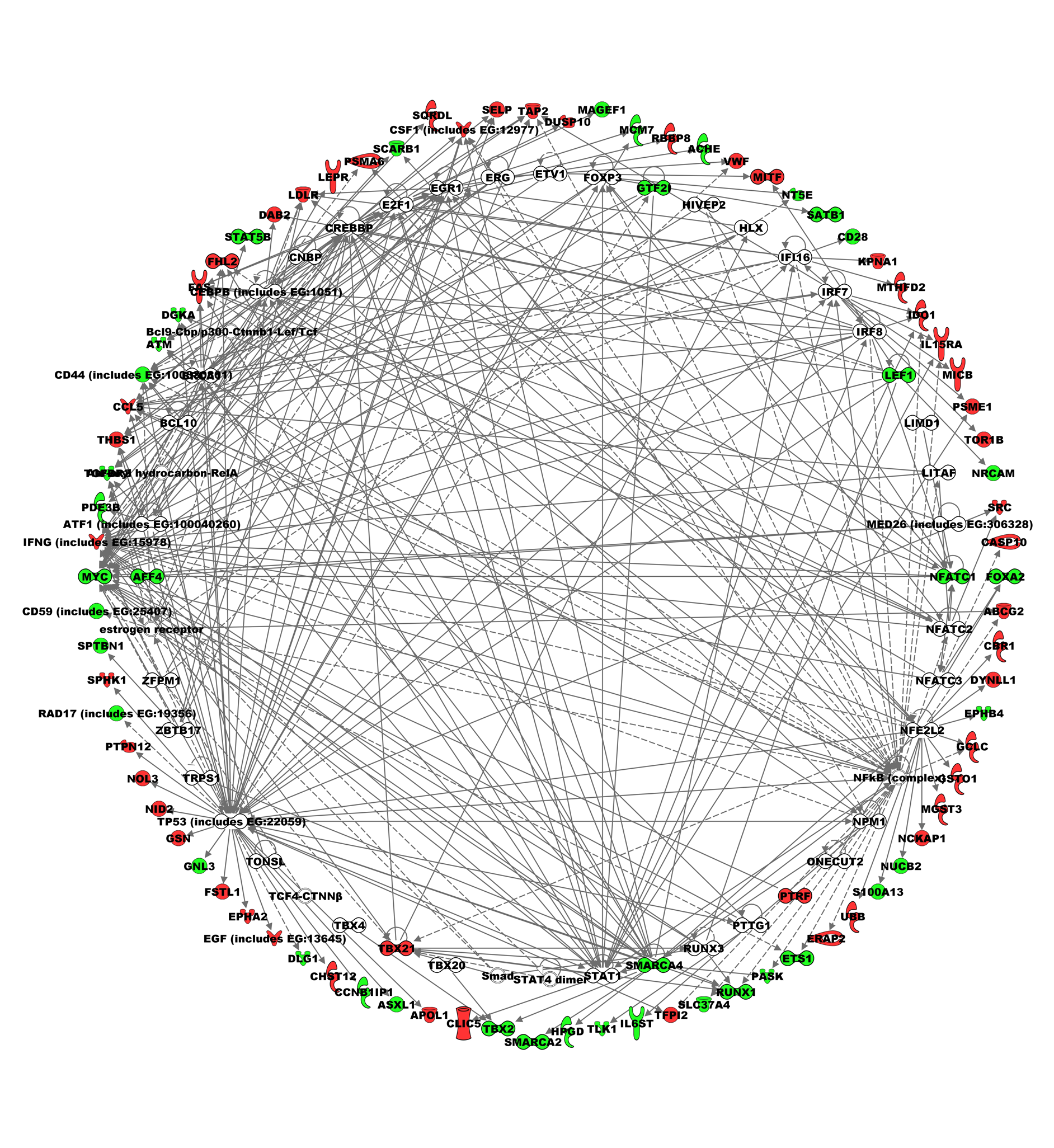

Supplement: Figure S1 — Transcription factors and corresponding genes operating in BMS. The relations between TFs and corresponding genes within the BMS signature were inferred using data-mining IPA software. The inner circle represents 49 predicted TFs that potentially could regulate the corresponding target genes within the BMS signature, represented in the external circle. Colored nodes present MIGs or TFs that significantly changed within the signature; red – over-expressed, green – down-expressed; white nodes represent TFs that were predicted (z-score) to be involved in the target genes regulation, while only seven colored TFs indeed changed their expression level within the BMS signature. (TIF) [file pone.0046871.s002.tif]
